# Effectiveness of Zinc Supplementation for Sepsis Treatment: A Single-Center Retrospective Observational Study

**DOI:** 10.3390/nu16172841

**Published:** 2024-08-25

**Authors:** Muneyoshi Kim, Takaaki Maruhashi, Yasushi Asari

**Affiliations:** Department of Emergency and Critical Care Medicine, Kitasato University School of Medicine; 1-15-1 Kitasato, Minami-ku, Sagamihara 252-0375, Japan

**Keywords:** zinc, trace elements, sepsis, intensive care, nutrition therapy

## Abstract

Background: Zinc plays an important role in sepsis; however, the effectiveness of zinc supplementation and the appropriate dose remain unclear. This study aimed to verify the effectiveness of zinc supplementation and the appropriate dose in patients with sepsis. Methods: This single-center retrospective observational study included 247 patients with sepsis from 1 April 2015 to 31 March 2023 who were receiving ventilatory management. The patients were divided into three groups according to the zinc supplementation dose: <15 mg, 15–50 mg, and ≥50 mg. Results: The <15 mg, 15–50 mg, and ≥50 mg groups had 28 (19%), six (21%), and 16 deaths (22%) at discharge, with no statistically significant difference (*p* = 0.36). No statistically significant differences were observed in the length of intensive care unit (ICU) stay (*p* = 0.06). A higher supplementation dose corresponded with a statistically significant increase in blood zinc concentration in the first week (38.5 ± 16.6 µg/dL, 58.8 ± 19.7 µg/dL, 74.2 ± 22.5 µg/dL, respectively; *p* < 0.01) but not in the second or third weeks (*p* = 0.08, 0.19, respectively). Conclusions: Zinc supplementation did not reduce the mortality rate or length of ICU stay or contribute to an increased serum zinc concentration. High-dose zinc supplementation may not be effective during acute sepsis.

## 1. Introduction

Zinc is the second most abundant metal in the body after iron and is an essential mineral that must be consumed through the diet to maintain its blood concentration level, since it cannot be synthesized by the body [1]. Zinc deficiency can result in growth disorders and wound healing deficiencies, owing to Zinc’s important role in DNA construction. Moreover, it is involved in T-cell activation, dendritic cell maturation, mast cell degranulation, and cytokine production, making it crucial for both acquired and natural immunity [2].

A previous retrospective study on severe burns reported that a higher serum zinc concentration corresponded with a shorter time to epithelization [3]. In recent years, the effects of zinc supplementation have garnered attention amidst the COVID-19 pandemic. A randomized controlled trial (RCT) on COVID-19 reported that the zinc administration group had a lower mortality rate (2.7%) and a lower intensive care unit (ICU) admission rate (approximately 6%) than the placebo group [4]. Various previous reports have indicated that zinc supplementation improves survival rates [5]. However, a previous observational study on head trauma reported that zinc supplementation showed no significant impact on the mortality rate [6]. In addition, a systematic review of the effects of zinc supplementation in ICUs [7] found no benefits from zinc supplementation.

The European Society for Clinical Nutrition and Metabolism (ESPEN) micronutrient guidelines recommend a zinc dosage of 10 mg/1500 kcal for enteral nutrition and 3–5 mg/day for parenteral nutrition [8]. However, the guidelines of the American Society for Parenteral and Enteral Nutrition and the Japanese Society for Parenteral and Enteral Nutrition Therapy do not indicate the need to supplement with zinc alone [9]. Therefore, the appropriate zinc supplementation dose for severely ill patients remains unclear.

Sepsis is characterized by systemic inflammation triggered by infections with microorganisms such as bacteria, and the mortality rate for patients with sepsis admitted to an ICU is approximately 30% [10]. Zinc’s crucial role in the outcome of sepsis has been hypothesized, owing to its involvement in the immune system and cytokine production. The 2021 Surviving Sepsis Campaign guidelines [11] set recommendations regarding trace element supplementation; however, the effectiveness of zinc supplementation and the appropriate supplementation dose were not discussed. We hypothesized that higher levels of zinc supplementation would normalize the serum zinc concentration and improve outcomes in sepsis. Therefore, this study aimed to verify the effectiveness of zinc supplementation and the appropriate dose based on data from previously reported sepsis cases at our facility.

## 2. Materials and Methods

### 2.1. Study Design and Setting

This was a single-center retrospective observational study of patients admitted to our facility from 1 April 2015 to 31 March 2023. This study was conducted in accordance with the Declaration of Helsinki and approved by the Kitasato University Hospital Review Board (Approval number: B23-026). Patient consent was waived, owing to the retrospective nature of the study. The patients were diagnosed with sepsis and received ventilatory management. The diagnosis of sepsis was based on the definition in Sepsis-3 [12], published in 2016. Cases before 2016 were reviewed via medical records to re-verify whether they met the diagnostic criteria. Patients whose blood zinc concentration changes could not be assessed due to death or discharge within a week after admission were excluded.

Our facility is a regional flagship hospital with approximately 1200 hospital beds, including 20 emergency-ICU (EICU) beds. The EICU was managed by intensive care physicians, and nutrition therapy was provided in accordance with the ESPEN guidelines. Nutrition was provided in a small amount via nasogastric tubes within 48 h of admission. Target calories were based on values measured using indirect calorimetry. In principle, permissive underfeeding was adopted at the start of nutrition therapy, and nutrition was gradually increased thereafter to achieve the target calorie intake within a week. The target protein intake was ≥1.2 g/kg. Additionally, the basic policy for enteral nutrition was to administer 125 mg of V CRESC^®^ (NUTRI Co., MIE, Yokkaichi, Japan) for trace elements from the start of nutrition therapy. Blood tests for nutritional indicators, including blood zinc concentration, were performed at the first visit and once a week thereafter. The requirement for zinc supplementation, other than V CRESC^®^, and the dose were determined at the discretion of attending physicians based on the blood zinc concentration.

### 2.2. Data Collection and Study Outcomes

First, the data of patients who received ventilator management were extracted from the ICU admission database. Subsequently, the data of patients diagnosed with sepsis were extracted. The following patient data were extracted from medical records: age; sex; weight; body mass index (BMI); presence or absence of underlying diseases; nutritional status assessment (CONUT score); severity (Acute Physiology and Chronic Health Evaluation II [APACHE-II] score, Sequential Organ Failure Assessment [SOFA] score) at ICU admission; mechanical ventilation days; requirement for renal replacement therapy; blood test findings (white blood cell count, lymphocyte count, platelet count, total bilirubin, creatinine, total protein, albumin, total cholesterol, C-reactive protein [CRP]) at first visit and at the first, second, and third weeks of admission; zinc supplementation dose; length of ICU stay; and outcomes at discharge. The zinc supplementation dose was calculated, with the zinc contents of 12 mg for V CRESC, 17 mg for Promac, 70 mg for zinc acetate hydrate, and 134.2 mg for zinc sulfate hydrate. These zinc supplementation products were orally and enterally administered. Zinc contained in intravenous formulations was not included in this study, owing to its negligible quantities in these formulations.

The participants were classified into three groups: the <15 mg group, the 15–50 mg group, and the ≥50 mg group. Patient outcomes at discharge were compared among the three groups as the primary outcome. Secondary outcomes included comparisons of the length of ICU stay and serum zinc concentration from the first to third weeks of admission among the three groups.

### 2.3. Statistical Analysis

Continuous variables were compared using the Mann–Whitney U test or Kruskal–Wallis test, as applicable. Data were presented as mean ± standard deviation. Dichotomous variables were presented as percentages and compared using Fisher’s exact test. Kaplan–Meier survival analysis was used to compare in-hospital mortality rates among the three groups at different zinc supplementation doses. Furthermore, multivariate analysis using logistic regression analysis was performed to evaluate the association between zinc supplementation dose and in-hospital mortality. Explanatory variables were selected using a stepwise method. Statistical significance was set at *p* < 0.05. All statistical analyses were performed using IBM SPSS Statistics ver. 26.

## 3. Results

Overall, 286 patients with sepsis received ventilator management during the study period. Of them, 39 met the exclusion criteria, and 247 were analyzed. The mean age of the included participants was 69.3 ± 14.3 years, with a sex ratio of 160:87. The most common sepsis focus was the digestive system in 108 patients (43.7%), followed by the lungs in 79 patients (32.0%), skin and soft tissues in 27 patients (10.9%), urinary tract in 17 patients (6.9%), and others in 16 patients (6.5%). The mean length of ICU stay was 18.1 ± 13.5 days, and the mean length of hospital stay was 53.3 ± 46.1 days, with 50 deaths at discharge (20%).

**Figure 1:**
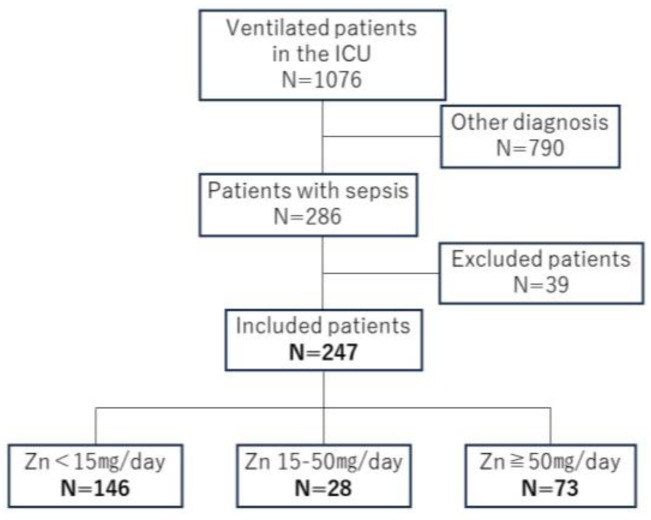
Patient inclusion flowchart.

The patients were classified into three groups based on the zinc supplementation dose: 146 patients in the <15 mg group, 28 patients in the 15–50 mg group, and 74 patients in the ≥50 mg group (Figure 1). A comparison of the three groups revealed no statistically significant difference in patient background characteristics, including age, weight, BMI, and infection focus (Table 1). Moreover, no significant differences were observed in the SOFA and APACHE-II scores among the three groups. Statistically significant differences were observed in the CONUT scores among the groups (9.8 ± 1.8 vs. 10.4 ± 1.7 vs. 10.3 ± 1.7; *p* = 0.03).

Of the 1076 patients on ventilator management in the ICU, 286 were diagnosed with sepsis. Overall, 247 patients were enrolled in this study, excluding 39 patients whose blood zinc concentration changes could not be assessed due to death or discharge within a week after admission.

A comparison of blood test findings at the first visit revealed no significant differences in white blood cells, lymphocyte count, platelet count, total bilirubin, creatinine, total protein, total cholesterol, and CRP levels. A statistically significant difference was only observed in albumin levels (2.0 ± 0.5 vs. 1.9 ± 0.5 vs. 1.8 ± 0.5; *p* < 0.01).

**Figure 2:**
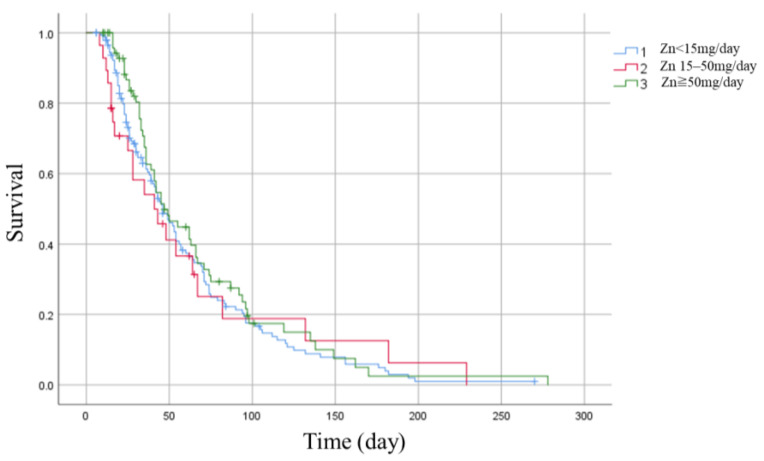
Kaplan–Meier curve.

The <15 mg group had 28 deaths at discharge (19%), the 15–50 mg group had six (21%), and the ≥50 mg group had 16 deaths (22%), with no statistically significant difference in the primary outcome (*p* = 0.36) (Figure 2).

No statistically significant difference was observed in survival rates among the three groups classified by zinc supplementation dose (<15 mg, 15–50 mg, ≥50 mg) (Table 2).

**Figure 3:**
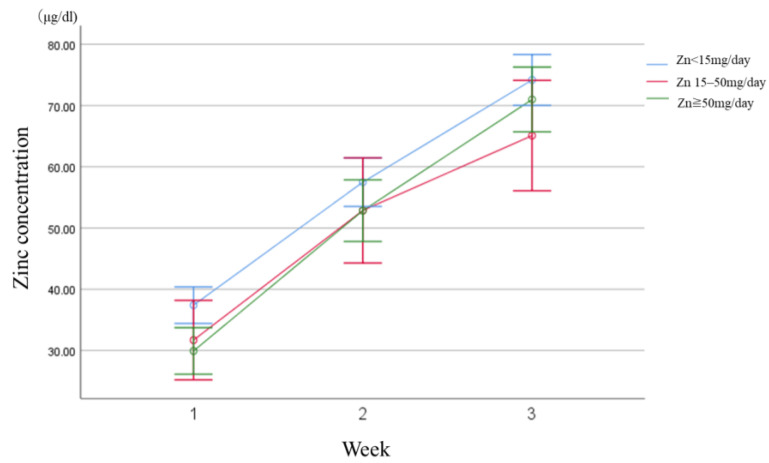
Changes in blood zinc concentration. A higher zinc supplementation dose corresponded with a higher blood concentration in the first week. However, no difference was observed among the three groups over time in the second (58.8 ± 19.7 µg/dL, 55.3 ± 27.0 µg/dL, and 53.6 ± 19.3 µg/dL [*p* = 0.08]) and third week (74.2 ± 22.5 µg/dL, 65.1 ± 20.3 µg/dL, and 71.0 ± 21.5 µg/dL [*p* = 0.19]).

In addition, no statistically significant differences were observed in the length of ICU stay, a secondary outcome, at 17.5 ± 13.4, 18.2 ± 17.0, and 19.5 ± 12.2 days, respectively (*p* = 0.06). Regarding blood zinc concentration changes after admission, a higher supplementation dose corresponded with a statistically significant increase in blood zinc concentration in the first week (38.5 ± 16.6 µg/dL, 58.8 ± 19.7 µg/dL, 74.2 ± 22.5 µg/dL, respectively; *p* < 0.01); however, no statistically significant differences were observed in the second and third weeks (*p* = 0.08, 0.19, respectively) (Figure 3).

A comparison of survival and death cases is shown in Table 3. Multivariate analysis with in-hospital mortality as the dependent variable revealed that the SOFA score and albumin level were associated with risk factor of survival (odds ratio = 1.103, 95% confidence interval: 1.008–1.207, *p* = 0.03, odds ratio = 0.482, 95% confidence interval: 0.240–0.969, *p* = 0.04, respectively). The dose of Zn supplementation was not an independent risk factor for survival.

## 4. Discussion

In this study, we investigated the effectiveness of zinc supplementation and the appropriate dose in patients with sepsis. Our findings showed that differences in the zinc supplementation dose did not influence the survival rate and blood zinc concentration in the acute phase of sepsis, possibly due to the pharmacokinetics of zinc during sepsis. Generally, 25–40% of consumed zinc is absorbed by the duodenum and jejunum into the body. Zinc binds to albumin and α2-macroglobulin in the blood and is stored in various organs via the liver. Regarding zinc distribution in tissues, 60% is found in the muscles, followed by 20–30% in bones and 8% in skin and hair. Two types of zinc transporters, namely ZIP (SLC30) and ZnT (SLC39), are present in the body. ZIP (SLC30) transports zinc from the outside of the cell into the cytoplasm, and ZnT (SLC39) maintains the homeostasis of the intracellular zinc concentration by secreting zinc from the cytoplasm to the outside of the cell and transporting it from the cytoplasm into intracellular organelles [13]. However, the pharmacokinetics of zinc are known to change during sepsis. A basic experiment using sepsis model mice showed a decline in the exogenous zinc uptake [14]. In addition, another study reported that the expression of SLC39A8 (a zinc importer) and the intracellular shift of zinc increased during sepsis, resulting in decreased serum zinc concentrations. This reaction is reported to be correlated with the increase in IL-8 [15]; therefore, various inflammatory cytokines alter the expression of zinc transporters during sepsis, resulting in the transfer of zinc from blood vessels into organs and cells, which decreases the serum zinc concentration.

Furthermore, impaired exogenous zinc utilization due to stress response caused by trauma or shock has been reported. Circulatory failure in mice subjected to traumatic stress results in a remarkable decline in the binding capacity of zinc to albumin [16]. This decreased binding capacity does not improve, even after restoration of the albumin concentration. A similar effect occurs in patients with severe sepsis, where zinc uptake may be reduced, despite exogenous supplementation. The results of previous studies and our study suggest that zinc may not be efficiently absorbed by the body and may not function effectively during sepsis, even if zinc supplementation is started in the invasive acute phase.

Moreover, excessive zinc supplementation may cause a decline in serum copper levels. Hypocupremia results in neutropenia and severe anemia, whereas severe hypocupremia results in neurological disorders because excessive zinc intake causes intestinal endothelial cells to produce metallothionein, which binds to zinc. Metallothionein is known to bind strongly to copper. When metallothionein is exfoliated from the inside of the intestinal lumen, the bound copper is concurrently excreted. A previous study reported that 9% of the patients prescribed zinc supplements presented with typical symptoms of copper deficiency [17]. In this report, 60% were supplemented with zinc at 135 mg/day. A supplementation dose of approximately 100–150 mg/day is likely to cause a decline in serum copper levels [18,19,20,21]. In this study, copper levels were not routinely measured; therefore, a comparison of copper levels by zinc supplementation dose could not be performed. However, iatrogenic hypocupremia caused by high-dose zinc supplementation may have affected the outcomes. All the zinc preparations that can be currently prescribed in Japan exceed the recommended daily intake dose (men: 11 mg/day; women: 9 mg/day), necessitating caution. Based on the abovementioned data, high-dose supplementation of zinc alone is ineffective in acute phases, such as sepsis, and supplementation with a small amount of trace elements, such as with V CRESC, may be necessary.

### Limitations

This study had certain limitations. First, this was a single-center retrospective observational study with a small sample size, which may have introduced bias. In addition, the zinc supplementation dose was determined at the discretion of attending physicians, owing to the lack of guidelines on zinc supplementation. Finally, zinc is measured in-house at our facility, and the results can be checked on the same day as that of sample collection and testing. However, copper testing is outsourced and requires time to obtain the results; therefore, the patients’ copper levels were not routinely measured. A high zinc supplementation dose may have caused hypocupremia and influenced the outcomes. The effectiveness of zinc supplementation and the appropriate supplementation dose must be verified through large-scale RCTs with matching patient backgrounds to address these limitations.

## 5. Conclusions

In this study, zinc supplementation for sepsis did not reduce the mortality rate or length of ICU stay or contribute to an increase in serum zinc concentration. Supplementation with high-dose zinc alone may not be effective in the acute phase of sepsis.

## Figures and Tables

**Table 1:** Sample background and clinical characteristics.

	<15 mg/Day	15–50 mg/Day	≥50 mg/Day	*p* Value
	N = 146	N = 28	N = 73	
Age (years)	68.9 ± 14.1	67.8 ± 16.8	70.6 ± 13.8	0.73 *
Sex (male) (%)	98 (67.1)	19 (67.9)	27 (37.0)	0.81 **
Weight (kg)	65.9 ± 15.8	58.6 ± 13.1	64.3 ± 15.7	0.12 *
Body mass index	24.7 ± 5.1	22.3 ± 3.4	24.9 ± 6.3	0.04 *
CONUT score	9.8 ± 1.8	10.4 ± 1.7	10.3 ± 1.7	0.03 *
Infection focus (%)				
Lung	55 (37.7)	7 (25.0)	17 (23.3)	0.07 **
Digestive organs	55 (37.7)	14 (50.0)	39 (53.4)	0.06 **
Urinary tract	14 (9.6)	1 (3.6)	2 (2.7)	0.15 **
Skin/Soft tissue	16 (11.0)	2 (7.1)	9 (12.3)	0.83 **
Others	6 (4.1)	4 (14.3)	6 (8.2)	0.09 **
Severity of ICU admission				
SOFA score	10.5 ± 3.8	8.7 ± 3.8	10.0 ± 4.1	0.07 *
APACHE-II score	31.7 ± 7.0	30.1 ± 7.1	30.1 ± 7.6	0.24 *
Total ventilation days	13.5 ± 16.4	17.1 ± 34.6	15.5 ± 17.6	0.23 *
Ventilation days >7 days	103 (70.5)	13 (46.4)	53 (72.6)	0.04 **
Renal replacement therapy	61 (41.8)	9 (32.1)	31 (42.5)	0.86 **
L/D on initial arrival (: normal range of our hospital)				
CRP (mg/dL; <0.14)	14.2 ± 11.2	14.5 ± 8.6	12.7 ± 9.2	0.61 *
Albumin (g/dL: 4.1–5.1)	2.03 ± 0.5	1.9 ± 0.5	1.8 ± 0.5	<0.01 *
WBC (×10^3^/µL: 3.3–8.6)	13.8 ± 11.1	12.7 ± 6.2	14.3 ± 7.1	0.23 *
Lymphocyte (/µL)	893.8 ± 937.8	654.1 ± 393.7	715.8 ± 445.8	0.20 *
Platelet (×10^4^/µL: 15.8–34.8)	16.5 ± 10.7	16.5 ± 8.7	16.5 ± 19.7	0.11 *
Total bilirubin (mg/dL: 0.4–1.5)	1.65 ± 2.5	2.1 ± 6.1	1.8 ± 2.7	0.55 *
Creatinine (mg/dL: 0.65–1.07)	1.58 ± 1.56	1.5 ± 1.1	1.5 ± 1.7	0.98 *
Total protein (g/dL: 6.6–8.1)	4.86 ± 0.78	4.5 ± 0.6	4.6 ± 0.7	0.17 *
Total cholesterol (mg/dL: 142–248)	118.2 ± 38.8	104.8 ± 34.3	117.0 ± 44.3	0.21 *

CONUT: Controlling Nutritional status, ICU: intensive care unit, SOFA: sequential organ failure assessment, APACHE: acute physiology and chronic health evaluation, L/D: laboratory data, CRP: C-reactive protein, WBC: white blood cell. * Kruskal–Wallis test, ** Fisher’s exact test.

**Table 2:** Study outcomes.

	<15 mg/Day	15–50 mg/Day	≥50 mg/Day	*p* Value
Primary outcome				
In-hospital mortality (%)	28 (19%)	6 (21%)	16 (22%)	0.36 **
Secondary outcome				
Length of ICU stay (day)	17.5 ± 13.4	18.2 ± 17.0	19.5 ± 12.2	0.06 *
Serum zinc concentration (µg/dL) (normal range of our hospital: 80–130)				
At the 1st week of admission	38.5 ± 16.6	58.8 ± 19.7	74.2 ± 22.5	<0.01 *
At the 2nd week of admission	58.8 ± 19.7	55.3 ± 27.0	53.6 ± 19.3	0.08 *
At the 3rd week of admission	74.2 ± 22.5	65.1 ± 20.3	71.0 ± 21.5	0.19 *

ICU: intensive care unit. * Kruskal–Wallis test, ** Fisher’s exact test.

**Table 3:** Comparison of characteristics between survival and death cases.

	Survival	Death	*p* Value
	N = 198	N = 49	
Age (year old)	68.6 ± 14.6	71.8 ± 12.9	0.16 *
Sex (male) (%)	124 (62.6)	19 (67.9)	0.05 **
Weight (kg)	63.5 ± 15.3	68.9 ± 16.2	0.04 *
Body mass index	24.1 ± 5.37	26.1 ± 5.1	0.01 *
CONUT score	9.9 ± 1.8	10.4 ± 1.6	0.04 *
Infection Focus (%)			
Lung	60 (30.3)	19 (38.8)	0.26 **
Digestive organs	91 (46.0)	17 (34.7)	0.16 **
Urinary tract	14 (7.0)	3 (6.1)	0.41 **
Skin/Soft tissue	22 (11.1)	5 (10.2)	0.86 **
Others	11 (5.6)	5 (10.2)	0.19 **
SOFA score	9.8 ± 3.7	11.7 ± 4.3	0.05 *
APACHE II score	30.6 ± 6.7	33 ± 8.	0.12 *
Total ventilation days (days)	13.3 ± 20.0	19.4 ± 16.9	0 *
Renal replacement therapy (%)	72 (36.3)	29 (59.1)	<0.01 **
Dose of zinc supplementation	46.8 ± 50.1	54.4 ± 55.9	0.39 *
Serum zinc concentration (µg/dL) (normal range of our hospital: 80–130)			
At the 1st week of admission	34.9 ± 15.1	36.9 ± 18.2	0.71 *
At the 2nd week of admission	58.2 ± 20.6	61.0 ± 19.3	0.19 *
At the 3rd week of admission	74.4 ± 21.8	61.7 ± 19.7	<0.01 *
Other L/D on initial arrival (normal range of our hospital)			
CRP (mg/dL; <0.14)	14.5 ± 10.5	10.9 ± 8.3	0.02 *
Albumin (g/dL: 4.1–5.1)	2.0 ± 0.5	1.8 ± 0.5	<0.01 *
WBC (×10^3^/µL: 3.3–8.6)	13.4 ± 9.9	15.7 ± 8.6	0.03 *
Lymphocyte (/µL)	833.9 ± 783.4	751.7 ± 657	0.17 *
Platelet (×10^4^/µL: 15.8–34.8)	16.5 ± 10.6	16.7 ± 22.1	0.02 *
Total bilirubin (mg/dL: 0.4–1.5)	1.3 ± 2.1	3.3 ± 5.3	<0.01 *
Creatinine (mg/dL: 0.65–1.07)	1.5 ± 1.5	1.9 ± 1.5	0.02 *
Total protein (g/dL: 6.6–8.1)	4.8 ± 0.8	4.7 ± 0.7	0.59 *
Total cholesterol (mg/dL: 142–248)	120.3 ± 40.0	106 ± 37.2	0.02 *

CONUT: Controlling Nutritional status, SOFA: sequential organ failure assessment, APACHE: acute physiology and chronic health evaluation, L/D: laboratory data, CRP: C-reactive protein, WBC: white blood cell. * Mann–Whitney U test, ** Fisher’s exact test.

## Data Availability

The datasets generated and analyzed during the current study are available from the corresponding author upon reasonable request due to privacy.

## References

[1] Lubna S., Ahmad R. (2023). Clinical and biochemical understanding of Zinc interaction during liver diseases: A paradigm shift. J. Trace Elem. Med. Biol..

[2] Hirano T., Murakami M., Fukada T., Nishida K., Yamasaki S., Suzuki T. (2008). Roles of zinc and zinc signaling in immunity: Zinc as an intracellular signaling molecule. Adv. Immunol..

[3] Olson L.M., Coffey R., Porter K., Thomas S., Bailey J.K., Jones L.M., Murphy C.V. (2000). The impact of serum zinc normalization on clinical outcomes in severe burn patients. Burns.

[4] Ben Abdallah S., Mhalla Y., Trabelsi I., Sekma A., Youssef R., Bel Haj Ali K., Ben Soltane H., Yacoubi H., Msolli M.A., Stambouli N. (2023). Twice-daily oral zinc in the treatment of patients with Coronavirus Disease 2019: A randomized double-blind controlled trial. Clin. Infect. Dis..

[5] Al Sulaiman K., Aljuhani O., Al Shaya A.I., Kharbosh A., Kensara R., Al Guwairy A., Alharbi A., Algarni R., Al Harbi S., Vishwakarma R. (2021). Evaluation of zinc sulfate as an adjunctive therapy in COVID-19 critically ill patients: A two center propensity-score matched study. Crit. Care.

[6] Khazdouz M., Mazidi M., Ehsaei M.R., Ferns G., Kengne A.P., Norouzy A.R. (2018). Impact of zinc supplementation on the clinical outcomes of patients with severe head trauma: A double-blind randomized clinical trial. J. Diet. Suppl..

[7] Gitte K.V., Thomas S.J., Karen L.E., Morten H.M., Thordis T., Anders P. (2023). Effects of magnesium, phosphate, or zinc supplementation in intensive care unit patients—A systematic review and meta-analysis. Acta Aaesthesiol. Scand..

[8] Berger M.M., Shenkin A., Schweinlin A., Amrein K., Augsburger M., Biesalski H.K., Bischoff S.C., Casaer M.P., Gundogan K., Lepp H.L. (2022). ESPEN micronutrient guideline. Clin. Nutr..

[9] McClave S.A., Taylor B.E., Martindale R.G., Warren M.M., Johnson D.R., Braunschweig C., McCarthy M.S., Davanos E., Rice T.W., Cresci G.A. (2016). Guidelines for the Provision and Assessment of Nutrition Support Therapy in the Adult Critically Ill Patient: Society of Critical Care Medicine (SCCM) and American Society for Parenteral and Enteral Nutrition (A.S.P.E.N.). JPEN J. Parenter. Enteral Nutr..

[10] Prescott H.C., Harrison D.A., Rowan K.M., Shankar-Hari M., Wunsch H. (2024). Temporal trends in mortality of critically ill patients with sepsis in the UK, 1988–2019. Am. J. Respir. Crit. Care Med..

[11] Evans L., Rhodes A., Alhazzani W., Antonelli M., Coopersmith C.M., French C., Machado F.R., Mcintyre L., Ostermann M., Prescott H.C. (2021). Surviving sepsis campaign: International guidelines for management of sepsis and septic shock 2021. Intensive Care Med..

[12] Singer M., Deutschman C.S., Seymour C.W., Shankar-Hari M., Annane D., Bauer M., Bellomo R., Bernard G.R., Chiche J.D., Coopersmith C.M. (2016). The Third International Consensus Definitions for Sepsis and Septic Shock (Sepsis-3). JAMA.

[13] Kodama H., Tanaka M., Naito Y., Katayama K., Moriyama M. (2020). Japan’s practical guidelines for zinc deficiency with a particular focus on taste disorders, inflammatory bowel disease, and liver cirrhosis. Int. J. Mol. Sci..

[14] Srinivas U., Ohlsson T., Hallstadius L., Hansson L., Abdulla M., Strand S.E., Jeppsson B. (1989). Organ sequestration of ^65^Zn during experimental sepsis. Clin. Nutr..

[15] Besecker B.Y., Exline M.C., Hollyfield J., Phillips G., Disilvestro R.A., Wewers M.D., Knoell D.L. (2011). A comparison of zinc metabolism, inflammation, and disease severity in critically ill infected and noninfected adults early after intensive care unit admission. Am. J. Clin. Nutr..

[16] Kelly E., Mathew J., Kohler J.E., Blass A.L., Soybel A.D. (2012). Hemorrhagic shock and surgical stress alter distribution of labile zinc within high- and low-molecular-weight plasma fractions. Shock.

[17] Duncan A., Yacoubian C., Watson N., Morrison I. (2015). The risk of copper deficiency in patients prescribed zinc supplements. J. Clin. Pathol..

[18] Forman W.B., Sheehan D., Cappelli S., Coffman B. (1990). Zinc abuse--an unsuspected cause of sideroblastic anemia. West. J. Med..

[19] Porter K.G., McMaster D., Elmes M.E., Love A.H. (1977). Anaemia and low serum-copper during zinc therapy. Lancet.

[20] Patterson W.P., Winkelmann M., Perry M.C. (1985). Zinc-induced copper deficiency: Megamineral sideroblastic anemia. Ann. Intern. Med..

[21] Igic P.G., Lee E., Harper W., Roach K.W. (2002). Toxic effects associated with consumption of zinc. Mayo Clin. Proc..

